# 854. Performance of a Nasopharyngeal-Based Host Gene Expression Classifier at Distinguishing Bacterial versus Viral Respiratory Infection

**DOI:** 10.1093/ofid/ofad500.899

**Published:** 2023-11-27

**Authors:** Jack G Anderson, Nicholas O’Grady, Champica K Bodinayake, Ajith Nagahawatte, Gaya B Wijayaratne, Nayani P Weerasinghe, Tianchen Sheng, Rachel Myers, Bradly P Nicholson, Thomas W Burke, Micah T McClain, Christopher W Woods, L Gayani Tillekeratne

**Affiliations:** Duke University, Durham, North Carolina; Duke University, Durham, North Carolina; University of Ruhuna, Galle, Southern Province, Sri Lanka; University of Ruhuna, Galle, Southern Province, Sri Lanka; University of Ruhuna, Galle, Southern Province, Sri Lanka; University of Ruhuna, Galle, Southern Province, Sri Lanka; Duke University Medical Center, Durham, NC; Duke University, Durham, North Carolina; Institute for Medical Research, Durham, North Carolina; Duke University, Durham, North Carolina; Duke University, Durham, North Carolina; Duke University, Durham, North Carolina; Duke University, Durham, North Carolina

## Abstract

**Background:**

Acute respiratory infections (ARI) present a diagnostic challenge due to overlapping clinical features of bacterial and viral infections, leading to unnecessary use of antibiotics. We have previously shown that a host-based blood mRNA expression test based on a 24-gene classifier has excellent performance differentiating bacterial and viral (B vs V) ARI. Since obtaining venous blood can be invasive and impractical in some settings, we examined the performance of this previously derived host mRNA test on nasopharyngeal (NP) samples.

**Methods:**

We enrolled patients presenting with ARI to a tertiary care hospital in Sri Lanka from November 2019 to October 2020 and collected blood samples in PAXgene RNA tubes paired with NP samples in RNAlater. We selected a convenient sample of 21 subjects with a confirmed bacterial or viral ARI, extracted RNA using commercially available kits, and measured 24 host mRNA transcripts using a multiplex RT-PCR-based assay optimized for venous blood (Biomeme Franklin M1, Philadelphia, PA). Due to missing values, one target was removed from the 24-gene classifier. Two models, one for each sample type, were built using elastic net logistic regression as implemented in the glmnet R package to classify B vs V infections. Nested leave-one-out cross-validation was used to conduct both parameter regularization (lambda) and predictions of bacterial probability, with alpha set to 0.01.

**Results:**

With 21 subjects [median age (IQR) 64 (56-66) years, 13 female, 8 male], the model trained on the PAXgene samples was able to correctly classify 8 of 9 bacterial and 8 of 12 viral ARIs using a 23 gene classifier, with an area under the curve (AUC) of 0.824 [confidence interval (CI) of 0.64-1]. Due to significantly different cycle thresholds in the normalizers, the NP sample set necessitated a second model that used 16 subjects [median age 61 (49-64) years, 10F, 6M]. This model correctly classified all 11 viral and 3 of 5 bacterial ARIs with an AUC of 0.818 (CI 0.57-1).

**Conclusion:**

These results suggest that a host mRNA expression test based on non-invasive sampling from the nasopharynx shows promise in classifying B vs V ARI. These preliminary findings need to be confirmed in a larger cohort of subjects to develop tests that can help reduce antibiotic overuse for ARI.

**Disclosures:**

**Thomas W. Burke, PhD**, Biomeme Inc: Advisor/Consultant|Biomeme Inc: Methods to diagnose and treat acute respiratory infections|Biomeme Inc: Ownership Interest **Micah T. McClain, MD, PhD**, Biomeme Inc: Methods to diagnose and treat acute respiratory infections **Christopher W. Woods, MD, MPH**, Biomeme Inc: Methods to diagnose and treat acute respiratory infections|Biomeme Inc: Chief Medical Consultant|Biomeme Inc: Ownership Interest

